# Vitamin A and vitamin B12 levels and coronary artery atherosclerosis: Univariate and multivariate Mendelian randomization analysis

**DOI:** 10.1097/MD.0000000000044244

**Published:** 2025-09-05

**Authors:** Luofei Huang, Jian Shi, Han Li, Quanzhi Lin

**Affiliations:** a Liuzhou Municipal Liutie Central Hospital, Liuzhou, Guangxi, China; b Department of Internal Medicine, The People’s Hospital of Laibin, Laibin, Guangxi, China; c Department of Internal Medicine, Liuzhou People’s Hospital, Liuzhou, Guangxi, China; d Department of Internal Medicine, The First Affiliated Hospital of Guangxi University of Science and Technology, Liuzhou, Guangxi, China.

**Keywords:** coronary artery atherosclerosis, genome-wide association studies, Mendelian randomization, vitamin A, vitamin B12

## Abstract

Coronary artery atherosclerosis (CAA) stands as a prominent etiological contributor to global cardiovascular morbidity and mortality. Its pathogenesis entails intricate interplays among genetic predisposition, environmental factors, and lifestyle determinants. Trace elements, though necessitated in minuscule quantities, have emerged as potential modulators of CAA progression, yet their exact impact remains unclear. We utilized Mendelian randomization (MR) analysis, employing genetic variants as instrumental variables to investigate the causal relationship between trace element levels and CAA. This study conducted a dual-sample MR analysis using data extracted from genome-wide association studies (GWAS) and the FinnGen database. To assess the statistical significance of these associations, we applied various MR statistical methods including MR-Egger, weighted median, and inverse variance weighted (IVW). Additionally, to further validate the robustness of our findings, multivariable MR analysis was performed. This approach allowed us to control for potential confounders, providing more precise causal inference results. The research findings reveal a significant negative correlation between vitamin A and the risk of CAA, indicating its role as a protective factor (according to the IVW method, odds ratio [OR] = 0.018, 95% confidence interval [CI] = 0.001–0.487, *P* = .017). Conversely, genetically predicted vitamin B12 shows a significant positive correlation with CAA risk, suggesting its role as a risk factor for CAA (OR = 1.268, 95% CI = 1.059–1.518, *P* = .01). However, in the multivariable regression analysis, both vitamin A and vitamin B12 remained significantly associated with CAA risk, with respective ORs of 0.020 (95% CI = 0.002–0.254, *P* = .003) and 1.252 (95% CI = 1.040–1.506, *P* = .018). This study elucidates the critical role of trace elements in the pathogenesis of CAA, providing a theoretical basis for personalized interventions and precision medicine. Further research could explore therapeutic strategies targeting trace element modulation to improve cardiovascular health.

## 1. Introduction

Coronary artery atherosclerosis (CAA) stands as a prominent etiological contributor to global cardiovascular morbidity and mortality.^[1]^ Its pathogenesis entails intricate interplays among genetic predisposition, environmental factors, and lifestyle determinants.^[2,3]^ The progression of CAA is characterized by the incremental deposition of lipids, inflammatory processes, and fibrotic alterations within the coronary vasculature, precipitating luminal constriction and subsequent ischemic events.^[4,5]^ Conventional therapeutic modalities encompassing lifestyle modifications, pharmacotherapy, and invasive interventions aim to assuage symptoms, impede disease advancement, and mitigate adverse prognostic outcomes associated with CAA.^[6–8]^

Trace elements, though necessitated in minuscule quantities, exert pivotal regulatory roles across diverse physiological processes and have emerged as potential modulators of CAA progression.^[9,10]^ Their intricate involvement in oxidative stress, endothelial functionality, and inflammatory cascades underscores their critical relevance in cardiovascular homeostasis.^[11]^ Dysregulation of trace element homeostasis has been implicated in the pathogenesis of CAA, with deficiencies or surfeits of certain elements exacerbating disease progression.^[12,13]^ Observational studies suggest an association between deficiencies in vitamins B12 and A and coronary artery diseases,^[14]^ while conversely, evidence suggests limited efficacy in supplementing antioxidant forms of vitamin A in coronary atherosclerosis.^[15]^ The study by Lonn et al indicates that vitamin B12 does not reduce the risk of major cardiovascular events in patients with vascular diseases.^[16]^

Mendelian randomization (MR) analysis provides a potent tool to delineate causal relationships between trace elements and CAA, leveraging genetic variants as instrumental variables (IVs) to emulate randomized controlled trials in observational studies.^[17]^ By harnessing the fortuitous assortment of genetic alleles during gamete formation in accordance with Mendel laws, MR analysis mitigates biases inherent in conventional observational studies, such as confounding and reverse causation.^[18]^ Furthermore, MR analysis capitalizes on the innate randomization of genetic variants at conception, furnishing robust estimates of causal effects.^[19]^ This methodological approach empowers researchers to scrutinize the impact of trace element levels on CAA risk, elucidating potential therapeutic targets and preventive strategies. Leveraging the MR framework furnishes unparalleled insights into the intricate interplay between trace elements and CAA,^[20]^ fostering the development of personalized interventions and precision medicine paradigms in cardiovascular care.

## 2. Materials and methods

### 2.1. Research design

In our investigation, we employed a dual-sample MR analysis approach to scrutinize the causal relationship between trace element levels and CAA. The MR causal inference relies on 3 fundamental assumptions. Firstly, the robust correlation between genetic variation and exposure variables is crucial.^[21]^ Secondly, it is imperative to ensure that potential confounders do not distort the relationship between genetic variation and the outcome (CAA).^[22]^ Lastly, we must ascertain that the pathway through which exposure (trace elements) affects the outcome is limited to genetic variation and not influenced by other pleiotropic effects.^[23]^ This stringent methodological framework ensures the reliability of our causal inferences, elucidating the role of trace elements in the pathogenesis of CAA.

### 2.2. Exposure and outcome data origins

The exposure and outcome data originate from the genome-wide association studies (GWAS) database (https://gwas.mrcieu.ac.uk/). For the exposure data, we included 15 trace elements, namely Copper (GWAS ID: ieu-a-1073), Selenium (GWAS ID: ieu-a-1077), and Zinc (GWAS ID: ieu-a-1079), sourced from the European population studied by Evans, with a sample size of 2603.^[24]^ Additionally, 12 other trace elements, including calcium (ukb-b-8951), carotene (ukb-b-16202), folate (ukb-b-11349), iron (ukb-b-20447), magnesium (ukb-b-7372), potassium (ukb-b-17881), vitamin A (ukb-b-9596), vitamin B12 (ukb-b-19524), vitamin B6 (ukb-b-7864), vitamin C (ukb-b-19390), vitamin D (ukb-b-18593), and vitamin E (ukb-b-6888), were sourced from the European population studied by BenElsworth, with a sample size of 64,979 and a number of ingle nucleotide polymorphisms (SNPs) totaling 9851,867. The CAA dataset was obtained from the FinnGen Research database, consisting of GWAS summary statistics from 51,589 cases and 343,079 controls.

### 2.3. MR and statistical analysis

We included SNPs with genome-wide significance (*P* < 5 × 10^−5^)^[25]^ and utilized linkage disequilibrium reference panels from the 1000 Genomes Project to ensure the independence of IVs. Specifically, we set an *R*^2^ threshold of < 0.001 within a distance of 100,000 kb.^[26]^ For MR analysis, we established an *F*-statistic threshold (*F* > 10) to identify and exclude weak IVs associated with each SNP.^[27]^ We employed various methods, including MR-Egger,^[28]^ weighted median,^[29]^ inverse variance weighted (IVW),^[30]^ simple mode,^[31]^ and weighted mode,^[32]^ to distinguish SNPs showing consistent directions of effect in odds ratio (OR) values. The MR-Egger method detects and adjusts for pleiotropic effects by incorporating an intercept term, making it suitable for scenarios where pleiotropy is present. The weighted median method assumes that at least 50% of the IVs are valid, providing robustness against invalid instruments. In contrast, the IVW method yields the most efficient causal estimates in the absence of pleiotropy. Specifically, MR-Egger is sensitive to pleiotropic effects, the weighted median approach is robust to invalid instruments, and IVW is most precise when pleiotropy is absent. However, IVW may introduce bias in the presence of pleiotropy or invalid instruments.

Results were meticulously analyzed based on OR values and supplemented with tests for pleiotropy and heterogeneity. Residual heterogeneity of the IVW model was assessed using Cochran *Q* test (*P* < .05). Additionally, MR-Egger intercept test was performed (*P* < .05). Data visualization encompassed comprehensive analysis using scatter plots, funnel plots, and forest plots. Scatter plots were used to demonstrate the elasticity of results to potential outliers. Funnel plots were utilized to confirm the robustness of associations and validate absence of heterogeneity. Finally, forest plots provided a visual representation of the interaction between IVs (SNPs) and study outcomes.

### 2.4. Multivariable MR analysis

We conducted a multivariable Mendelian randomization (MVMR)^[33]^ analysis using the IVW method. This analysis assumes all genetic variants serve as “valid” IVs, meaning the effect of SNPs on ACC outcomes is solely mediated through their impact on exposure/risk factors. The objective of this analysis is to delineate the genetic relationships between exposure variables and ascertain their associations with outcomes.

## 3. Results

### 3.1. Univariate MR analysis

**Figure 1.**
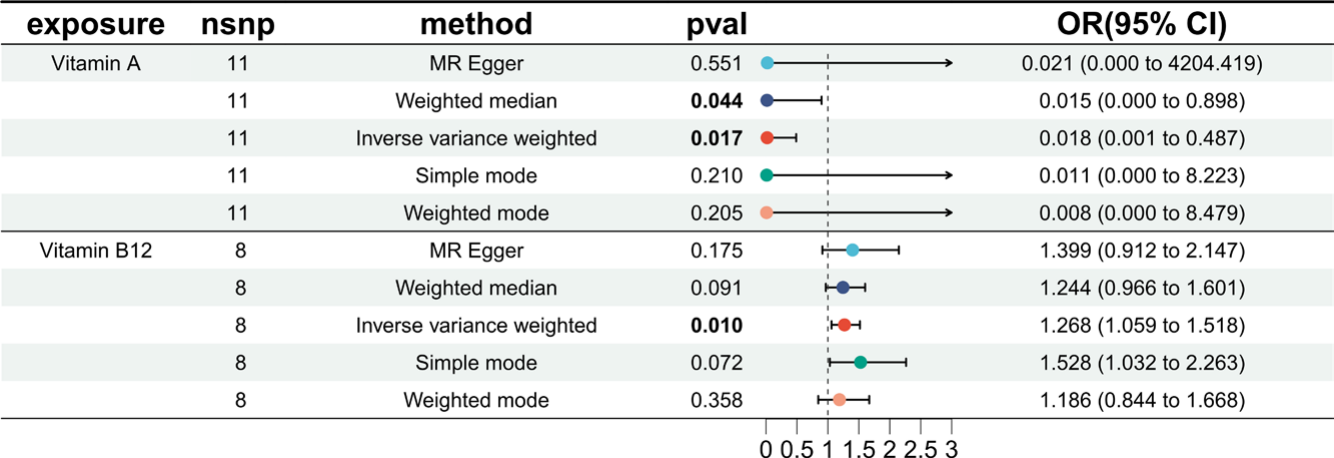
Forest plot of the causal relationship between trace elements and CAA. CAA = coronary artery atherosclerosis, CI = confidence interval, IVW = inverse variance weighted, OR = odds ratio.

All retained SNPs exhibited *F*-statistics exceeding 10, indicating a strong association between the selected IVs of trace elements and CAA as shown in Table S1 (Supplemental Digital Content, https://links.lww.com/MD/P824). Consequently, by excluding weak IVs, the reliability of the findings in this study was enhanced. In this analysis, trace elements data from the GWAS database were used as the exposure, with outcomes for CAA derived from the FinnGen database, facilitating a genetic analysis. Based on genetic predictions, Vitamin A demonstrated a significant negative correlation with CAA risk, acting as a protective factor against CAA (according to the IVW method, OR = 0.018, 95% confidence interval [CI] = 0.001–0.487, *P* = .017) (Fig. 1). indicating a 98.2% reduction in CAA risk per unit increase in vitamin A levels. This aligns with its known antioxidant properties, which mitigate oxidative stress and endothelial dysfunction, key drivers of atherosclerosis. Conversely, genetically predicted Vitamin B12 showed a significant positive correlation with CAA risk, indicating its role as a risk factor for CAA (OR = 1.268, 95% CI = 1.059–1.518, *P* = .01). This paradoxical finding may reflect B12’s role in homocysteine metabolism, where genetic variants could influence pathways beyond homocysteine reduction. Primary MR analyses, including a leave-one-out sensitivity chart (Fig. S1, Supplementary Digital Content, https://links.lww.com/MD/P823), scatter plot (Fig. S2, Supplementary Digital Content, https://links.lww.com/MD/P823), funnel plot (Fig. S3, Supplementary Digital Content, https://links.lww.com/MD/P823), and forest plot (Fig. S4, Supplementary Digital Content, https://links.lww.com/MD/P823), revealed no significant evidence of horizontal pleiotropy as indicated by the MR-Egger intercept. Tests for heterogeneity (Table S2, Supplemental Digital Content, https://links.lww.com/MD/P824) and pleiotropy (Table S3, Supplemental Digital Content, https://links.lww.com/MD/P824) within the analysis demonstrated the absence of both.

### 3.2. MVMR analysis

**Figure 2.**
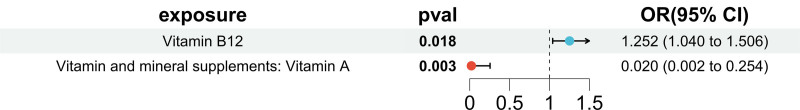
Forest diagram of multivariate Mendelian causal relationship between trace elements and CAA. CAA = coronary artery atherosclerosis, CI = confidence interval, IVW = inverse variance weighted, OR = odds ratio.

The MVMR analysis demonstrated that the protective effect of vitamin A against CAA persisted significantly, with an OR of 0.020 (95% CI: 0.002–0.254, *P* = .003). This effect size was moderately higher than that observed in the univariate analysis, suggesting that vitamin A exerts its protective role independent of other influencing factors. Notably, the positive association between vitamin B12 and CAA remained statistically significant, with an OR of 1.252 (95% CI: 1.040–1.506, *P* = .018), indicating that its role as a risk determinant is unaffected by other trace elements (Fig. 2).

## 4. Discussion

The results of our dual-sample MR analysis underscore the complex and nuanced roles of trace elements in the pathogenesis and progression CAA. Specifically, our findings highlight the differential impact of vitamins A and B12 on CAA risk, which aligns with emerging literature on the significance of micronutrient homeostasis in cardiovascular health.^[34,35]^ Vitamin A confers a protective role against CAA, exhibiting a significant negative correlation with CAA risk. This observation is consistent with previous studies, which identify vitamin A as a potent antioxidant capable of reducing the generation of free radicals. These radicals compromise cell membranes, including those of the endothelial cells in blood vessels, thus triggering inflammatory responses and vascular damage.^[36,37]^ By attenuating oxidative stress, vitamin A protects coronary arteries from the adverse effects of oxidized LDL cholesterol, a pivotal element in the development of atherosclerotic plaques.^[38]^ Moreover, vitamin A enhances the function of vascular endothelial cells and maintains the normal physiological state of the vasculature. Endothelial dysfunction is an early indicator of CAA progression, and maintaining endothelial health is essential for preventing inflammation and thrombosis. Additionally, vitamin A modulates the immune system by influencing the differentiation and activity of immune cells, thereby reducing inflammatory responses to the vascular wall. Inflammation plays a critical role in the advancement of CAA, with immune cells actively involved in plaque formation and progression.^[39]^ Contrary to expectations, our research findings indicate a positive correlation between vitamin B12 levels and the risk of CAA, suggesting a complex role for this nutrient in cardiovascular pathophysiology. Vitamin B12, a water-soluble vitamin, is crucial for the production of red blood cells, the maintenance of neurological function, and DNA synthesis.^[40,41]^ Furthermore, it plays a pivotal role in the metabolism of homocysteine, an amino acid whose elevated levels are associated with an increased risk of cardiovascular diseases due to its potential to promote inflammation and the development of atherosclerosis within the arteries.^[42,43]^ Vitamin B12 facilitates the conversion of homocysteine into methionine, thereby reducing its plasma concentrations.^[44,45]^ The accumulation of homocysteine is considered a risk factor for atherosclerosis, particularly when it co-occurs with inflammation of the arterial wall and endothelial dysfunction.^[46]^ Adequate intake of Vitamin B12, especially when combined with folic acid and Vitamin B6, is thought to prevent the progression of atherosclerosis primarily by lowering homocysteine levels.^[47,48]^ However, our study suggests that under certain genetic backgrounds, normal or elevated levels of Vitamin B12 may still contribute to disease progression, underscoring the necessity for a balanced approach to nutritional intake. Our study leverages the robustness of MR analysis, which allows for the assessment of causal relationships in observational data by using genetic variants as proxies for modifiable exposures.^[49,50]^ This approach is particularly beneficial in elucidating the role of nutrients, such as trace elements, which are influenced by both genetic makeup and dietary intake. The use of dual-sample MR further enhances the reliability of our findings by utilizing separate samples for estimating the associations of genetic instruments with exposure and outcome, thereby reducing sample overlap bias.^[51]^

From a clinical application perspective, the findings of this study hold certain guiding significance. Given the protective effect of vitamin A against CAA, in high-risk populations for coronary heart disease, it is reasonable to consider appropriate vitamin A supplementation interventions. Regarding vitamin B12, which serves as a risk factor for CAA, in clinical examinations, more attention should be paid to the vitamin B12 levels of coronary heart disease patients. If a patient has an elevated vitamin B12 level, it may be necessary to adjust the relevant treatment plan to reduce the risk of CAA. In clinical practice, incorporating trace element testing into the integrated metabolic risk score risk-scoring system can contribute to a more accurate assessment of patients’ conditions.^[52,53]^ This enables stratified management of patients and the development of more targeted treatment plans. Apart from vitamins A and B12, other elements such as copper, selenium, and zinc, although not showing a significant association with CAA in this analysis, do not necessarily play no role in the occurrence and development of CAA. In some previous studies, copper was found to be involved in the body’s redox reactions, and its imbalance may affect the function of vascular endothelial cells.^[54]^ Selenium, as an antioxidant, is crucial for maintaining the normal function of the cardiovascular system, and low selenium levels are associated with an increased risk of cardiovascular diseases. Zinc plays roles in cell metabolism and immune regulation and has potential links to inflammatory responses and vascular remodeling.^[55]^ The lack of significant associations in this study may be due to the limited sample size, which failed to detect these subtle effects, or differences in detection methods, resulting in inaccurate assessments of trace element levels.

While our MR analysis supports a causal role of vitamin A and B12 in CAA, the underlying genetic mechanisms remain to be fully elucidated. Future studies should investigate interactions between these vitamins and key genes implicated in CAA pathogenesis, such as those involved in oxidative stress (e.g., SOD1, CAT), lipid metabolism (e.g., APOE, LDLR), and homocysteine regulation (e.g., MTHFR, CBS). Integrating multi-omics data (e.g., transcriptomics or epigenetics) with genetic variants could clarify whether the observed effects are mediated through specific molecular pathways. Such research would advance precision nutrition strategies for CAA prevention by identifying genetic subgroups that may benefit most from vitamin A supplementation or require careful monitoring of B12 levels.

## 5. Limitations and future directions

Nevertheless, the interpretation of our findings must consider potential limitations inherent in MR studies. The assumption that genetic variants are reliably associated with trace element levels and influence CAA exclusively through these levels might oversimplify the complex interactions and pleiotropic effects that characterize biological systems. Furthermore, the external validity of our results may be influenced by the European ancestry of our study population, potentially limiting the generalizability to other ethnic groups. Our study paves the way for future research to explore the modulation of trace element levels as a therapeutic strategy for CAA. Given the protective role of Vitamin A and the risk-associated profile of Vitamin B12, further studies could investigate the optimal levels of these and other trace elements to prevent or mitigate CAA. Additionally, exploring the genetic determinants of individual responses to trace element supplementation could enhance personalized nutritional interventions in cardiovascular risk management. MVMR should also be employed more extensively to dissect the direct and indirect effects of trace elements, considering the potential confounders and mediators in their relationship with CAA. Such analyses will help clarify the pathways through which these nutrients exert their effects, supporting the development of targeted therapies and dietary recommendations.

## 6. Conclusion

In conclusion, our MR study demonstrates that genetically predicted vitamin A is protective against CAA (OR = 0.018), while vitamin B12 shows a risk-increasing effect (OR = 1.268). These findings suggest distinct roles of these vitamins in CAA pathogenesis, potentially through oxidative stress and homocysteine metabolism pathways. Our results highlight the importance of considering trace element levels in cardiovascular prevention strategies

## Acknowledgments

I sincerely appreciate the shared public database resources, which have been invaluable to my research and enhanced the quality of this study.

## Author contributions

**Conceptualization:** Luofei Huang, Quanzhi Lin.

**Data curation:** Luofei Huang, Quanzhi Lin.

**Formal analysis:** Luofei Huang, Han Li, Quanzhi Lin.

**Funding acquisition:** Quanzhi Lin.

**Investigation:** Han Li.

**Methodology:** Han Li.

**Software:** Quanzhi Lin.

**Writing – original draft:** Luofei Huang.

## Supplementary Material



## References

[1] Taqueti VR Di Carli MF. Coronary microvascular disease pathogenic mechanisms and therapeutic options: JACC state-of-the-art review. J Am Coll Cardiol. 2018;72:2625–41. 30466521 10.1016/j.jacc.2018.09.042 PMC6296779

[2] Libby P Theroux P. Pathophysiology of coronary artery disease. Circulation. 2005;111:3481–8. 15983262 10.1161/CIRCULATIONAHA.105.537878

[3] Ghattas A Griffiths HR Devitt A Lip GYH Shantsila E. Monocytes in coronary artery disease and atherosclerosis: where are we now? J Am Coll Cardiol. 2013;62:1541–51. 23973684 10.1016/j.jacc.2013.07.043

[4] Medina-Leyte DJ Zepeda-García O Domínguez-Pérez M González-Garrido A Villarreal-Molina T Jacobo-Albavera L. Endothelial dysfunction, inflammation and coronary artery disease: potential biomarkers and promising therapeutical approaches. Int J Mol Sci. 2021;22:3850. 33917744 10.3390/ijms22083850 PMC8068178

[5] Milutinović A Šuput D Zorc-Pleskovič R. Pathogenesis of atherosclerosis in the tunica intima, media, and adventitia of coronary arteries: an updated review. Bosn J Basic Med Sci. 2020;20:21–30. 31465719 10.17305/bjbms.2019.4320 PMC7029210

[6] Peña-Duque MA Romero-Ibarra JL Gaxiola-Macías MBA Arias-Sánchez EA. Coronary atherosclerosis and interventional cardiology. Arch Med Res. 2015;46:372–8. 26117516 10.1016/j.arcmed.2015.06.005

[7] Boudoulas KD Triposciadis F Geleris P Boudoulas H. Coronary atherosclerosis: pathophysiologic basis for diagnosis and management. Prog Cardiovasc Dis. 2016;58:676–92. 27091673 10.1016/j.pcad.2016.04.003

[8] Parsons C Agasthi P Mookadam F Arsanjani R. Reversal of coronary atherosclerosis: Role of life style and medical management. Trends Cardiovasc Med. 2018;28:524–31. 29807666 10.1016/j.tcm.2018.05.002

[9] Aalbers TG Houtman JP. Relationships between trace elements and atherosclerosis. Sci Total Environ. 1985;43:255–83. 4012298 10.1016/0048-9697(85)90133-0

[10] Cebi A Kaya Y Gungor H. Trace elements, heavy metals and vitamin levels in patients with coronary artery disease. Int J Med Sci. 2011;8:456–60. 21850195 10.7150/ijms.8.456 PMC3156992

[11] Qayyum R Schulman P. Iron and atherosclerosis. Clin Cardiol. 2005;28:119–22. 15813617

[12] Dabravolski SA Sukhorukov VN Melnichenko AA Khotina VA Orekhov AN. The role of selenium in atherosclerosis development, progression, prevention and treatment. Biomedicines. 2023;11:2010. 37509649 10.3390/biomedicines11072010 PMC10377679

[13] Meng H Wang Y Ruan J. Decreased iron ion concentrations in the peripheral blood correlate with coronary atherosclerosis. Nutrients. 2022;14:319. 35057500 10.3390/nu14020319 PMC8781549

[14] Riccioni G Bucciarelli T D'Orazio N. Plasma antioxidants and asymptomatic carotid atherosclerotic disease. Ann Nutr Metab. 2008;53:86–90. 18936536 10.1159/000164691

[15] McQuillan BM Hung J Beilby JP Nidorf M Thompson PL. Antioxidant vitamins and the risk of carotid atherosclerosis. The Perth Carotid Ultrasound Disease Assessment study (CUDAS). J Am Coll Cardiol. 2001;38:1788–94. 11738275 10.1016/s0735-1097(01)01676-x

[16] Lonn E Yusuf S Arnold MJ; Heart Outcomes Prevention Evaluation (HOPE) 2 Investigators. Homocysteine lowering with folic acid and B vitamins in vascular disease. N Engl J Med. 2006;354:1567–77. 16531613 10.1056/NEJMoa060900

[17] Birney E. Mendelian randomization. Cold Spring Harb Perspect Med. 2022;12:a041302. 34872952 10.1101/cshperspect.a041302 PMC9121891

[18] Sekula P Del Greco M F Pattaro C Köttgen A. Mendelian randomization as an approach to assess causality using observational data. J Am Soc Nephrol. 2016;27:3253–65. 27486138 10.1681/ASN.2016010098 PMC5084898

[19] Bowden J Holmes MV. Meta-analysis and mendelian randomization: a review. Res Synth Methods. 2019;10:486–96. 30861319 10.1002/jrsm.1346 PMC6973275

[20] Larsson SC Butterworth AS Burgess S. Mendelian randomization for cardiovascular diseases: principles and applications. Eur Heart J. 2023;44:4913–24. 37935836 10.1093/eurheartj/ehad736 PMC10719501

[21] Yeung CHC Schooling CM. Systemic inflammatory regulators and risk of Alzheimer’s disease: a bidirectional Mendelian-randomization study. Int J Epidemiol. 2021;50:829–40. 33313759 10.1093/ije/dyaa241

[22] Davies NM Howe LJ Brumpton B Havdahl A Evans DM Davey Smith G. Within family Mendelian randomization studies. Hum Mol Genet. 2019;28:R170–9. 31647093 10.1093/hmg/ddz204

[23] Zhou J Ye Z Wei P. Effect of basal metabolic rate on osteoporosis: a Mendelian randomization study. Front Public Health. 2023;11:1096519. 36817914 10.3389/fpubh.2023.1096519 PMC9929187

[24] Evans DM Zhu G Dy V. Genome-wide association study identifies loci affecting blood copper, selenium and zinc. Hum Mol Genet. 2013;22:3998–4006. 23720494 10.1093/hmg/ddt239 PMC3766178

[25] Burgess S Thompson SG. Multivariable Mendelian randomization: the use of pleiotropic genetic variants to estimate causal effects. Am J Epidemiol. 2015;181:251–60. 25632051 10.1093/aje/kwu283 PMC4325677

[26] Auton A Brooks LD Durbin RM; 1000 Genomes Project Consortium. A global reference for human genetic variation. Nature. 2015;526:68–74. 26432245 10.1038/nature15393 PMC4750478

[27] Burgess S Thompson SG; CRP CHD Genetics Collaboration. Avoiding bias from weak instruments in Mendelian randomization studies. Int J Epidemiol. 2011;40:755–64. 21414999 10.1093/ije/dyr036

[28] Bowden J Davey Smith G Burgess S. Mendelian randomization with invalid instruments: effect estimation and bias detection through Egger regression. Int J Epidemiol. 2015;44:512–25. 26050253 10.1093/ije/dyv080 PMC4469799

[29] Bowden J Davey Smith G Haycock PC Burgess S. Consistent estimation in mendelian randomization with some invalid instruments using a weighted median estimator. Genet Epidemiol. 2016;40:304–14. 27061298 10.1002/gepi.21965 PMC4849733

[30] Burgess S Small DS Thompson SG. A review of instrumental variable estimators for Mendelian randomization. Stat Methods Med Res. 2017;26:2333–55. 26282889 10.1177/0962280215597579 PMC5642006

[31] Wu F Huang Y Hu J Shao Z. Mendelian randomization study of inflammatory bowel disease and bone mineral density. BMC Med. 2020;18:312. 33167994 10.1186/s12916-020-01778-5 PMC7654011

[32] Zhang D Hu Y Guo W. Mendelian randomization study reveals a causal relationship between rheumatoid arthritis and risk for pre-eclampsia. Front Immunol. 2022;13:1080980. 36578485 10.3389/fimmu.2022.1080980 PMC9790901

[33] Richardson TG Sanderson E Palmer TM. Evaluating the relationship between circulating lipoprotein lipids and apolipoproteins with risk of coronary heart disease: a multivariable Mendelian randomisation analysis. PLoS Med. 2020;17:e1003062. 32203549 10.1371/journal.pmed.1003062 PMC7089422

[34] Yuan S Mason AM Carter P Burgess S Larsson SC. Homocysteine, B vitamins, and cardiovascular disease: a Mendelian randomization study. BMC Med. 2021;19:97. 33888102 10.1186/s12916-021-01977-8 PMC8063383

[35] Gidlöf AC Ocaya P Krivospitskaya O Sirsjö A. Vitamin A: a drug for prevention of restenosis/reocclusion after percutaneous coronary intervention? Clin Sci (Lond). 2008;114:19–25. 18047466 10.1042/CS20070090

[36] Tinkel J Hassanain H Khouri SJ. Cardiovascular antioxidant therapy: a review of supplements, pharmacotherapies, and mechanisms. Cardiol Rev. 2012;20:77–83. 22293859 10.1097/CRD.0b013e31823dbbad

[37] Riccioni G Mancini B Di Ilio E Bucciarelli T D'Orazio N. Protective effect of lycopene in cardiovascular disease. Eur Rev Med Pharmacol Sci. 2008;12:183–90. 18700690

[38] Gaziano JM. Antioxidant vitamins and coronary artery disease risk. Am J Med. 1994;97:18S–21S; discussion 22S. 10.1016/0002-9343(94)90294-1 8085582

[39] Diaz MN Frei B Vita JA Keaney JF. Antioxidants and atherosclerotic heart disease. N Engl J Med. 1997;337:408–16. 9241131 10.1056/NEJM199708073370607

[40] O’Leary F Samman S. Vitamin B12 in health and disease. Nutrients. 2010;2:299–316. 22254022 10.3390/nu2030299 PMC3257642

[41] Benites-Zapata VA Ignacio-Cconchoy FL Ulloque-Badaracco JR. Vitamin B12 levels in thyroid disorders: a systematic review and meta-analysis. Front Endocrinol (Lausanne). 2023;14:1070592. 36909313 10.3389/fendo.2023.1070592 PMC9994182

[42] Mayer EL Jacobsen DW Robinson K. Homocysteine and coronary atherosclerosis. J Am Coll Cardiol. 1996;27:517–27. 8606260 10.1016/0735-1097(95)00508-0

[43] Langman LJ Cole DE. Homocysteine. Crit Rev Clin Lab Sci. 1999;36:365–406. 10486705 10.1080/10408369991239231

[44] Mahalle N Kulkarni MV Garg MK Naik SS. Vitamin B12 deficiency and hyperhomocysteinemia as correlates of cardiovascular risk factors in Indian subjects with coronary artery disease. J Cardiol. 2013;61:289–94. 23473764 10.1016/j.jjcc.2012.11.009

[45] Siri PW Verhoef P Kok FJ. Vitamins B6, B12, and folate: association with plasma total homocysteine and risk of coronary atherosclerosis. J Am Coll Nutr. 1998;17:435–41. 9791839 10.1080/07315724.1998.10718790

[46] Schroecksnadel K Frick B Winkler C Leblhuber F Wirleitner B Fuchs D. Hyperhomocysteinemia and immune activation. Clin Chem Lab Med. 2003;41:1438–43. 14656023 10.1515/CCLM.2003.221

[47] Hodis HN Mack WJ Dustin L; BVAIT Research Group. High-dose B vitamin supplementation and progression of subclinical atherosclerosis: a randomized controlled trial. Stroke. 2009;40:730–6. 19118243 10.1161/STROKEAHA.108.526798 PMC2701290

[48] Newman PE. Can reduced folic acid and vitamin B12 levels cause deficient DNA methylation producing mutations which initiate atherosclerosis? Med Hypotheses. 1999;53:421–4. 10616044 10.1054/mehy.1998.0794

[49] Verduijn M Siegerink B Jager KJ Zoccali C Dekker FW. Mendelian randomization: use of genetics to enable causal inference in observational studies. Nephrol Dial Transplant. 2010;25:1394–8. 20190244 10.1093/ndt/gfq098

[50] Sheehan NA Meng S Didelez V. Mendelian randomisation: a tool for assessing causality in observational epidemiology. Methods Mol Biol. 2011;713:153–66. 21153618 10.1007/978-1-60327-416-6_12

[51] Sheehan NA Didelez V. Epidemiology, genetic epidemiology and Mendelian randomisation: more need than ever to attend to detail. Hum Genet. 2020;139:121–36. 31134333 10.1007/s00439-019-02027-3 PMC6942032

[52] Keskin M Kaya A Tatlisu MA. The effect of serum potassium level on in-hospital and long-term mortality in ST elevation myocardial infarction. Int J Cardiol. 2016;221:505–10. 27414730 10.1016/j.ijcard.2016.07.024

[53] Mert İlker H Faysal S Ahmet Çağdaş Y Murat S Tufan C. Prognostic value of intermountain risk score for short- and long-term mortality in patients with cardiogenic shock. Coron Artery Dis. 2023;34:154–9. 36720024 10.1097/MCA.0000000000001219

[54] Parsanathan R. Copper’s dual role: unravelling the link between copper homeostasis, cuproptosis, and cardiovascular diseases. Hypertens Res. 2024;47:1440–2. 38467792 10.1038/s41440-024-01636-4

[55] Gać P Czerwińska K Macek P. The importance of selenium and zinc deficiency in cardiovascular disorders. Environ Toxicol Pharmacol. 2021;82:103553. 33238203 10.1016/j.etap.2020.103553

