# Supplementary material for: Vitamin A and vitamin B12 levels and coronary artery atherosclerosis: Univariate and multivariate Mendelian randomization analysis
Source: Medicine (Baltimore). 2025 Sep 5;104(36):e44244. doi: 10.1097/MD.0000000000044244 (PMC12419359; doi:10.1097/MD.0000000000044244)

Supplementary Figure S1:Mendelian randomization Causal Sensitivity Analysis Diagram.


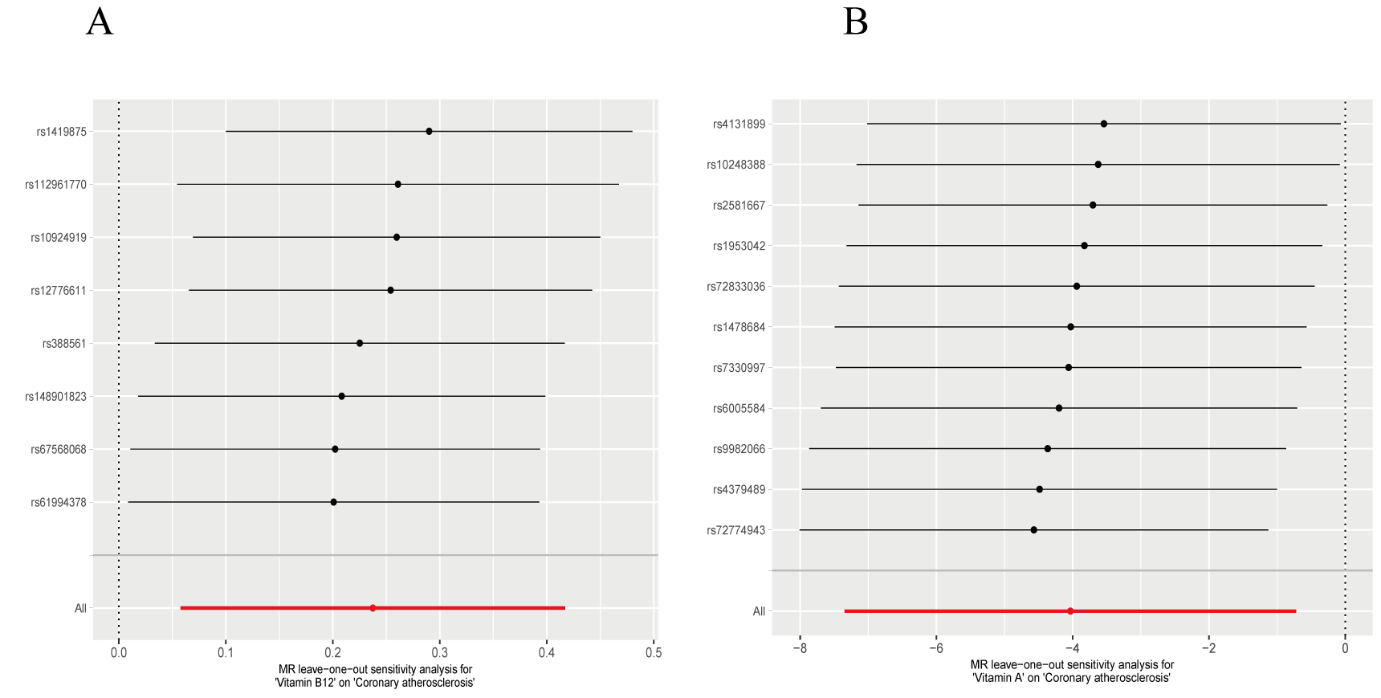


Supplementary Figure S2: Mendelian randomization Causal Scatter Plots.


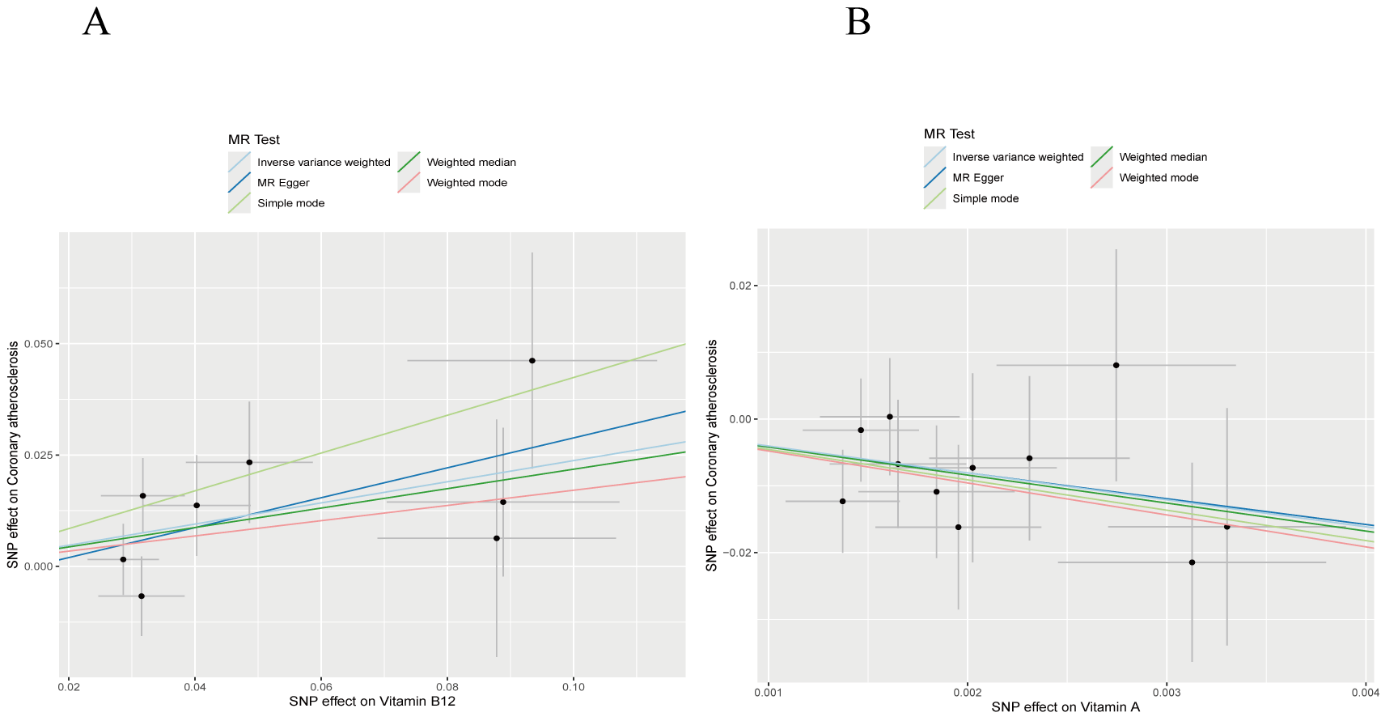


Supplementary Figure S3: Funnel plot for Mendelian randomization causal analysis.


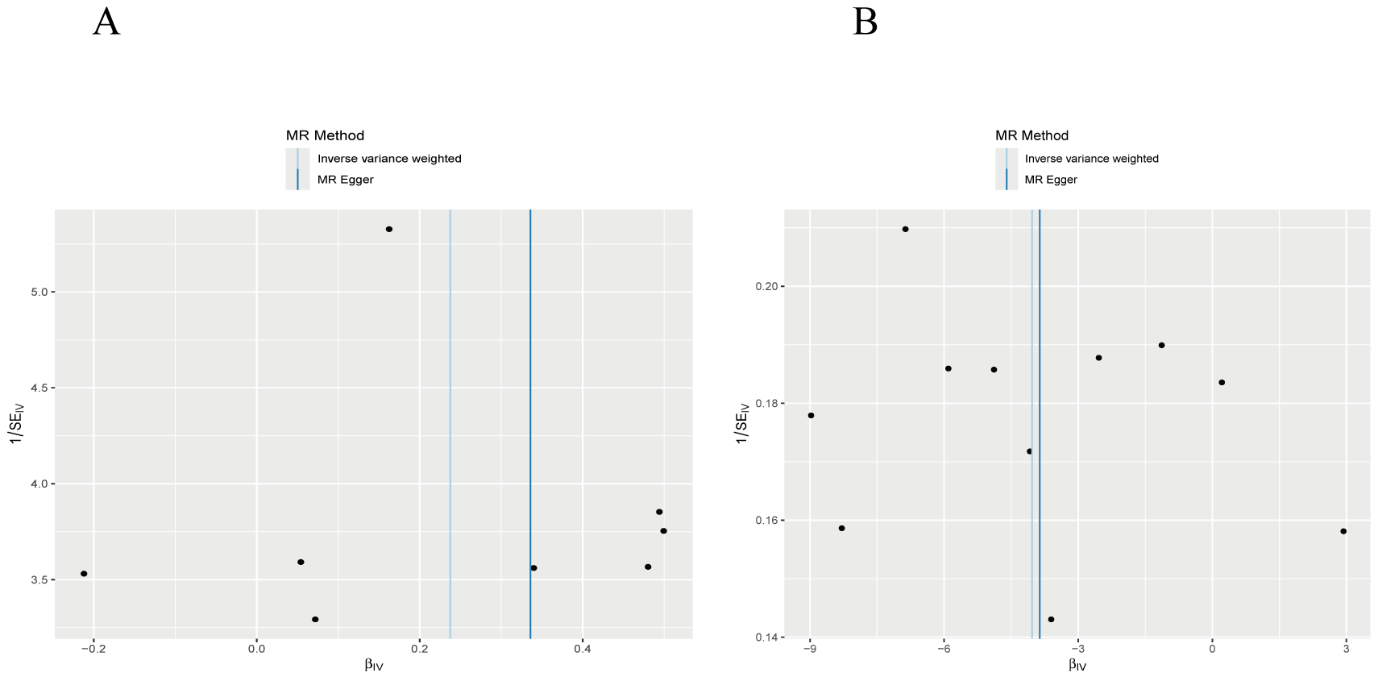


Supplementary Figure S4: Forest plot for Mendelian randomization causal analysis.


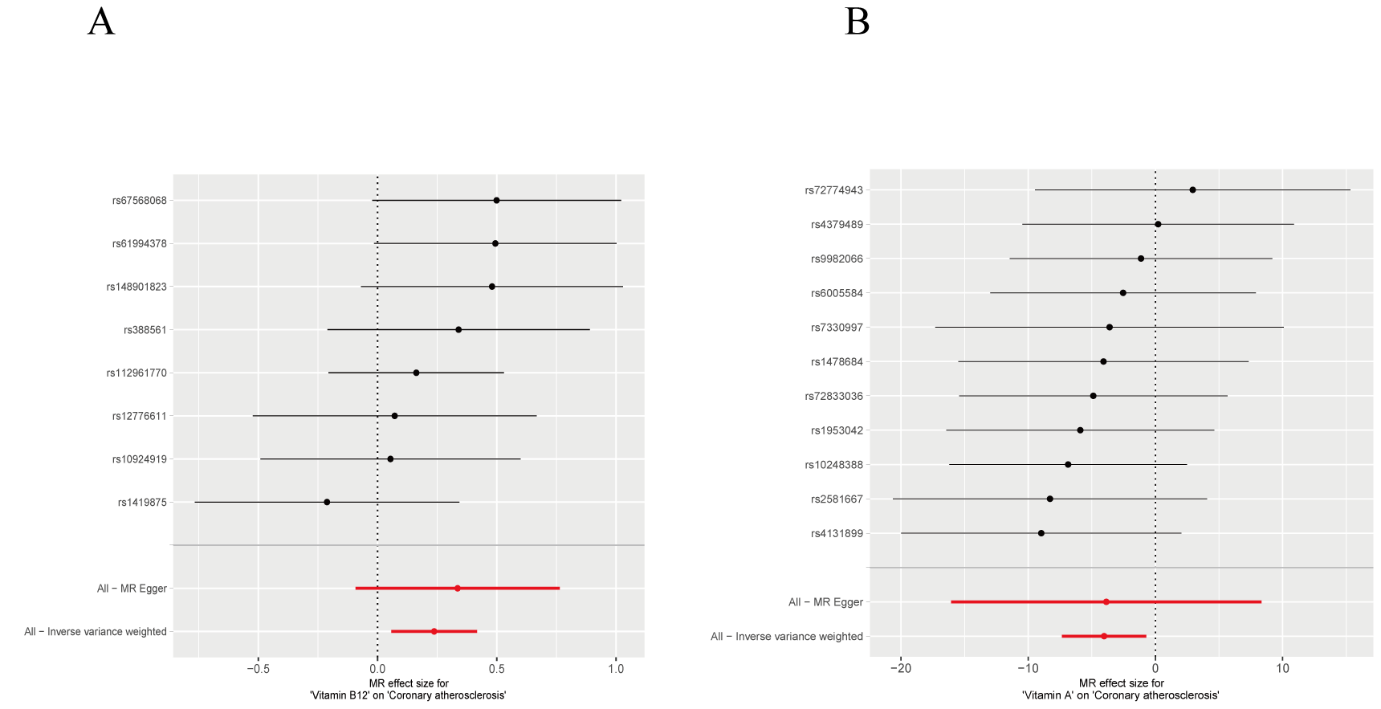

Supplement: Supplementary file 2 [file medi-104-e44244-s002.docx]
